# PD-1^+^CD8^+^ T Cells Proximal to PD-L1^+^CD68^+^ Macrophages Are Associated with Poor Prognosis in Pancreatic Ductal Adenocarcinoma Patients

**DOI:** 10.3390/cancers15051389

**Published:** 2023-02-22

**Authors:** Xiaobao Yang, Guanzheng Wang, Yue Song, Tongtao Zhuang, Yifei Li, Yujie Xie, Xuefeng Fei, Yanan Zhao, Dakang Xu, Yiqun Hu

**Affiliations:** 1 Department of Laboratory Medicine, Ruijin Hospital, Shanghai Jiao Tong University School of Medicine, Shanghai 200025, China; 2 Medical Technology Department, Qiqihar Medical University, Qiqihar 161000, China; 3 Xiangya School of Medicine, Central South University, Changsha 410013, China

**Keywords:** multiplexed IHC image cytometry, spatial analysis, PD-1^+^CD8^+^T cells, PD-L1^+^CD68^+^ TAMs, pancreatic ductal adenocarcinoma

## Abstract

**Simple Summary:**

The current methods for assessing tumor microenvironments (TMEs) using cellular markers and cell density-based assays do not identify the primitive phenotypes of single cells with multilineage selectivity or functional or cellular spatial information in tissues. Here, we combined the strategy of multiplex IHC imaging and cytometry-based cell quantification to assess multiple lineage-selective and functional phenotypic biomarkers and deeply dissect the state of immune complexity in the TME in association with clinical outcomes. Our findings showed that the percentage of CD8^+^ T cells expressing the T cell exhaustion marker PD-1 and/or the high expression of the checkpoint PD-L1 among CD68^+^ cells was associated with poor prognosis. The prognostic value of this combined approach is greater than that of lymphocyte and myeloid cell density analyses. Furthermore, a spatial analysis revealed that the abundance of PD-L1^+^CD68^+^ tumor-associated macrophages correlated with PD-1^+^CD8^+^ T cell infiltration levels. Our findings further strengthen the evidence for applying IHC imaging and cytometry-based cell analyses in precision medicine.

**Abstract:**

Immune complexity status in the TME has been linked to clinical outcomes in pancreatic ductal adenocarcinoma (PDAC) patients. TME assessments with current cell marker and cell density-based analyses do not identify the original phenotypes of single cells with multilineage selectivity, the functional status of the cells, or cellular spatial information in the tissues. Here, we describe a method that circumvents these problems. The combined strategy of multiplexed IHC with computational image cytometry and multiparameter cytometric quantification allows us to assess multiple lineage-selective and functional phenotypic biomarkers in the TME. Our study revealed that the percentage of CD8^+^ T lymphoid cells expressing the T cell exhaustion marker PD-1 and the high expression of the checkpoint PD-L1 in CD68^+^ cells are associated with a poor prognosis. The prognostic value of this combined approach is more significant than that of lymphoid and myeloid cell density analyses. In addition, a spatial analysis revealed a correlation between the abundance of PD-L1^+^CD68^+^ tumor-associated macrophages and PD-1^+^CD8^+^T cell infiltration, indicating pro-tumor immunity associated with a poor prognosis. These data highlight the implications of practical monitoring for understanding the complexity of immune cells in situ. Digital imaging and multiparameter cytometric processing of cell phenotypes in the TME and tissue architecture can reveal biomarkers and assessment parameters for patient stratification.

## 1. Introduction

The PDAC TME is highly heterogeneous and composed of stromal cells and immune cells, including T cells, macrophages, and natural killer (NK) cells. Immunosuppression is supported by preventing T cell infiltration into tumor tissues or inhibiting T cell killing functions [1,2,3]. In turn, immunosuppressive macrophages are recruited or the expression of the immune checkpoint (IC) molecules programmed cell death 1 (PD-1) and PD-ligand 1 (PD-L1) is upregulated, which are both common immune mechanisms in PDAC [4,5]. IC molecules promote tumor evasion by modifying immune cells, e.g., by inactivating immune cells and suppressing the immune response to tumors [6,7]. Therefore, the phenotype of immune cells in tumors, including cell subtypes, functional polarization, and spatial distribution, influences cancer patients’ prognoses and responses to PD-1/PD-L1-targeted immunotherapy.

The potential role of PD-1 in the maintenance of peripheral tolerance by negatively regulating T cell responses to antigenic stimulation and suppressing antitumor immunity has been reported in previous studies [8]. PD-L1 expression is a potential biomarker of immunotherapy response, and blocking the PD-1/PD-L1 interaction is important for reactivating T cell antitumor activities [9,10]. The PD-1/PD-L1 interaction is key in regulating T cell activation and expansion by suppressing T cell responses [11,12]. Since PD-1- and PD-L1-expressing cells and immune cells are proximal to and interact with tumor cells, the immunosuppressive and immunostimulatory mechanisms controlling antitumor immunity demonstrate a delicate and dynamic balance [13,14,15,16]. Characterizing the PD-1/PD-L1 axis to understand the mechanism of PD-L1^+^ macrophages and PD-1^+^CD8^+^ T cells migrating to the tumor site or the effect of the increasing immunosuppressive cellular components can provide insight into the complex interplay of immune and heterogeneous tumor cells in the TME [17]. Here, we aimed to investigate the involvement of PD-1/PD-L1-expressing immune cells in the TME to assess PDAC patients’ prognosis.

The immune cell heterogeneity in the TME has been intensively studied using the multiplex immunohistochemistry (mIHC) technique, which can characterize immune infiltration in tumor tissues with multiple biomarkers simultaneously stained on a single formalin-fixed paraffin-embedded (FFPE) slide. However, the current evaluation of the TME using single-cell markers and cell density-based assays cannot assess single-cell-based phenotypes with multiple lineage selectivity, functional status, or spatial relationship among individual cell types [18]. Thus, we combined mIHC with computational imaging and multiparametric flow cytometry quantification, which allowed us to assess multiple lineage-selective and functional phenotypic biomarkers in the TME. Image cytometry is a multiparameter cytometric approach that uses the quantification of fluorescence intensities based on single-cell segmentation. The single-cell data includes cell size, compactness, location, and fluorescence intensity per cell per marker. This quantification analysis was applied to multiple biomarkers in mIHC images, and the lymphoid or myeloid cells were assessed based on hierarchical gating strategies. Importantly, image cytometry enables the assessment of multiple lineage-selective and functional phenotypic biomarkers and in-depth analysis of spatial characteristics based on cell location and tissue context information in the TME. Combining mIHC with computational imaging and multiparametric flow cytometry quantification allowed us to assess multiple lineage-selective and functional phenotypic biomarkers in the TME. We applied mIHC and image-based flow cytometry, a novel and powerful technique that provides subcellular parameter information, to investigate the percentage of CD8^+^ T lymphoid cells expressing the T cell exhaustion marker PD-1 and the percentage of CD68^+^ tumor-associated macrophages (TAMs) expressing the checkpoint PD-L1 in PDAC. We also assessed the spatial distribution, prognostic value, and correlation between the proximity of PD-1^+^CD8^+^ T cells and PD-L1^+^CD68^+^ macrophages in the TME. We found that PD-1^+^CD8^+^ T cells and PD-L1^+^CD68^+^ TAMs coexisted in the PDAC TME. Evaluating the percentage of lymphoid CD8^+^ T cells expressing the T cell exhaustion marker PD-1 or the percentage of CD68^+^ cells expressing a high level of PD-L1 may serve as a better independent prognostic indicator for PDAC than assessing the density of tumor-infiltrating cells, which involves using a single marker to indicate an immune cell. Furthermore, the infiltration of a proportion of PD-1^+^ CD8^+^ T cells increased with the proportion of PD-L1^+^ CD68^+^ TAMs. These findings may provide new strategies and indications for developing optimal immunotherapy regimens for PDAC patients.

## 2. Materials and Methods

### 2.1. Patient Cohort and Tissue Microarray Construction

The human PDAC specimens were obtained from Ruijin Hospital, Shanghai Jiao Tong University School of Medicine (Shanghai, China), and a written informed consent was obtained from all participants. The study was approved by the human ethics committees of Ruijin Hospital, Shanghai Jiao Tong University School of Medicine (ID: 2021–207). The human tissue specimens were processed using FFPE. Based on the H&E staining results, which were examined by a pathologist, 170 cancer or adjacent normal FFPE tissue samples were punched and arranged in tissue microarray (TMA) blocks. A total of 80 tumor and adjacent normal tissue samples were paired, and an additional 10 tumor tissues were included. After discarding the incomplete tissues, the final tumor and adjacent normal dataset comprised 84 tumor and 73 adjacent normal samples. The clinical sample information of every patient is detailed in Supplementary Table S1, and Supplementary Table S2 shows the patients’ basic characteristics. Standard H&E staining was performed on the TMA sections using standard methods to further validate the tissue histology (Supplementary Figure S1). The diameter of each block core used in this TMA assessment was 1.5 mm.

### 2.2. Multiplex IHC Staining and Image Acquisition

We used the Ultivue UltiMapper Immuno 8 kit to conduct the mIHC staining according to the manufacturer’s instructions (#ULT30801, Ultivue, Cambridge, MA, USA) [19]. Paraffin-embedded sections were heated in an oven at 60 °C for 1 h, after which they were deparaffinized with xylene and rehydrated using a gradient of ethanol solutions. An antigen retrieval was performed in an EDTA (pH 9) buffer (Akoya Biosciences, Menlo Park, CA, USA), and the antibody diluent contained in the kit was used to block the binding of nonspecific antibodies. The commercialized primary antibodies used were pre-designed panels for identifying specific cells in the TME and included anti-CD3 (clone BC33), anti-CD8α (clone C8/144B), anti-FOXP3 (clone 236A/E7), anti-CD68 (clone KP-1), anti-PD-1 (clone CAL20), anti-PD-L1 (clone 73–10), and anti-PanCK/SOX10 (clone AE1/AE3 and BC34) antibodies. All antibodies were diluted at a ratio of 1:100 in antibody diluent and combined. The sections were then incubated in the antibody mixture for 1 h. After applying a pre-amplification mix and an amplification enzyme solution to detect antibody staining, the tissues were incubated with a nuclear counterstain solution, and the first-round fluorescent probe solution was used to detect CD8, PD-1, PD-L1, and CD68. A coverslip was then mounted over the tissue chip using the ProLong Gold Antifade Mountant (Thermo Fisher, Carlsbad, CA, USA). The sample was loaded onto the Vectra Polaris automated quantitative pathology imaging system (Akoya Biosciences), and whole-slide scanning captured the first-round images at a magnification of 20×. After acquiring the first round of images, an exchange solution was used to remove the fluorescent probes. A second round of staining was then conducted (CD3, FOXP3, and pan-keratin (PanCK)/SOX10), and the images were captured as described above.

### 2.3. Image Analysis

The HALO image analysis platform (Indica Labs, v3.3.2541.345) was used for image overlay, tissue segmentation, and cell phenotype analysis. The positive thresholds for each marker were set based on the nuclear (DAPI and FOXP3) or cytoplasmic (CD3, CD8, CD68, PD-1, PD-L1, and PanCK/SOX10) staining intensities and were examined across all tissue samples. The combined results for cell counts, densities, and percentages were exported for further analyses and for the generation of graphic images using a previously described method [20].

### 2.4. Flow Cytometry-like Workflow

The data were exported from the HALO software into a flow cytometry file (.fcs) format for the flow cytometry-like workflow. The single-cell data, including cell location and fluorescence intensity per cell per marker, were exported to the FCS Express 7 software (De Novo Software, Glendale, CA, USA) for this analysis. For each specimen, the marker expression was gated based on the fluorescence intensity of the corresponding negative control slide. The R software was used to construct scatter plots, which provided intuitive visual results of the cells gated by the FCS Express 7 software.

### 2.5. Spatial Analysis

The data collected on all of the evaluated markers in each cell and the (x, y) locations of the cells within the tissue specimens were stored in the HALO software for further spatial analyses. The proximity of the PD-1^+^ or PD-1^−^ CD8^+^ T cells to the PD-L1^+^ or PD-L1^−^ CD68^+^ macrophages was analyzed using the HALO Spatial Analysis module. In this module, the cells were defined by phenotype, and a spatial plot was generated for each sample. The proximity analysis tool was used to assess the distance between the cells and the percentage of PD-1^+^/PD-1^-^CD8^+^ T cells within 0–100 µm (number of bands = 10) of the PD-L1^+^CD68^+^ or PD-L1^-^CD68^+^ cells.

### 2.6. Survival Analysis

The median was used as a cutoff value to classify the patients into high- and low-expression groups. The patient survival analysis was performed in GraphPad Prism 9 (GraphPad, Inc., San Diego, CA, USA). The 1-year receiver operating curves (ROCs) for age, sex, stage, and cluster were plotted, and the areas under the curves (AUCs) of the ROC curves were calculated. A multivariate regression analysis was applied to evaluate the correlation between the overall survival (OS) outcomes and the age, sex, stage, and cluster information using the survival package in the R software. The HR was calculated and expressed as a forest plot.

### 2.7. Statistical Analysis

Statistical analyses were performed using GraphPad Prism 9. An unpaired, two-tailed Student’s *t*-test was used to determine statistically significant differences in the unpaired data. The correlations between the cell densities obtained using the flow cytometry-like workflow and HALO platforms or between the cell populations were evaluated using simple linear regression analyses. Multivariate Cox regression models were used to analyze the independent prognostic factors and immune markers. The overall survival outcomes among the subgroups were estimated by the Kaplan–Meier method, and the significant differences were assessed using log-rank tests. A *p* value of < 0.05 was considered to indicate significance (*: *p* < 0.05; **: *p* < 0.01; ***: *p* < 0.001).

## 3. Results

### 3.1. PDAC Cell Phenotyping with High-Multiplex InSituPlex DNA Barcoding and Antibody Staining

**Figure 1:**
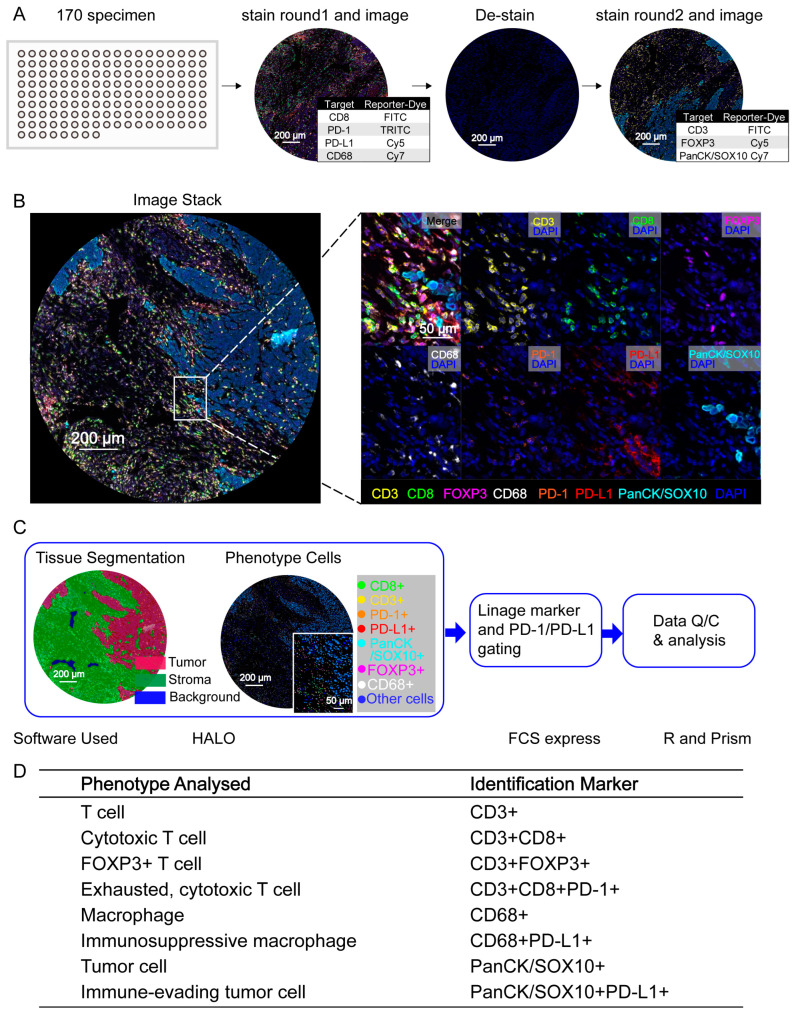
Characterization of cell phenotypes in PDAC tissues by InSituPlex multiplex staining technology. (**A**) A two-round multiplex staining assay and whole-slide digital scanning were performed in PDAC TMA, and the representative multispectral images (MSIs) of one TMA core are shown. (**B**) Simultaneous assessment of all markers using the “registration configure” functional module of the HALO software. The representative images show multiple staining patterns in the PDAC tissues. PanCK/SOX10 (a tumor cell marker) is shown in cyan; CD3^+^ T cells are shown in yellow; CD8^+^ cells are shown in green; FOXP3^+^ cells are shown in rose red; CD68^+^ cells are shown in white. The orange color indicates PD-1^+^ cells, and the red color indicates PD-L1^+^ cells. (**C**) The initial analysis was conducted using the HALO software. The tissues were segmented into tumor (red), stromal (green), and background (blue) regions based on DAPI^+^ and PanCK/SOX10^+^ cells. The cells were then distinguished into different types according to the identified markers. For the image cytometry analysis, the data were exported to the FCS Express 7 software to gate specific cell populations, and R and Prism were implemented for further analysis and data visualization. (**D**) Cell phenotypes identified by hierarchical gating of lineage-selective and functional biomarkers during image cytometry analysis of mIHC staining.

An FFPE tissue microarray comprising 80 paired tumors and adjacent normal tissue samples and 10 additional PDAC tumor tissue samples (a total of 170 tissue samples) was created to sufficiently and appropriately audit the complex and dynamic microenvironments that influenced the phenotypes of resident and infiltrating leukocytes in tumors with preserved geographic distribution. We established a two-round assay with each panel containing 3–4 biomarkers, including seven distinct epitopes of lymphoid and myeloid lineage cells and tumor epitopes (Figure 1A). The lymphoid biomarker panel depicts CD3^+^ T cells, CD3^+^CD8^+^ cytotoxic T cells, and CD3^+^FOXP3^+^ regulatory T cells (Tregs), while the myeloid biomarker panel shows CD68^+^ macrophages; the tumor cells were stained for PanCK/SOX10. Multispectral imaging followed by spectral separation and image overlay allow for the simultaneous assessment of all markers in the same slide tissue sample (Figure 1B). In addition, we performed the flow cytometry-like workflow described above. In this approach, an image analysis was conducted by transferring the resultant unmixed multilayer TIFF images into the HALO software for tissue and cell segmentation. The resultant single-cell data, which comprised cell location and fluorescence intensity per cell per marker, were exported to the FCS Express software for further analysis. The lineage assignment implicated the functional status of PD-1 or PD-L1 in immune cells (Figure 1C). Therefore, distinct cell phenotypes based on the markers described above were identified, and we quantitatively assessed six immune cell populations and two tumor cell populations (Figure 1D).

### 3.2. Lymphoid or Myeloid Density Alone Was Not Clearly Associated with Differences in Overall Survival Outcomes

**Figure 2:**
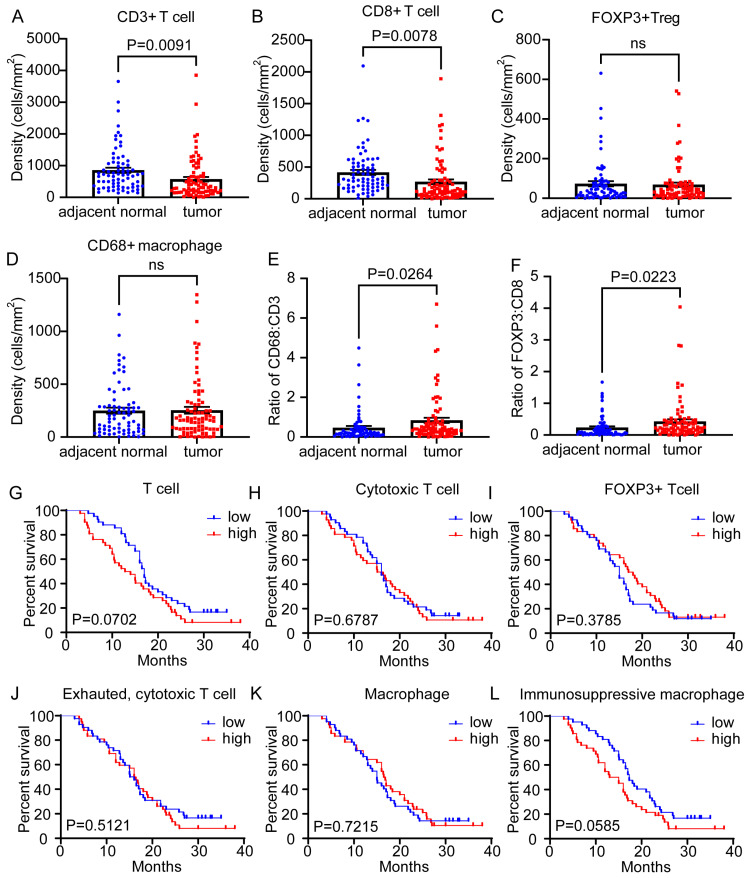
The analyzed phenotypes demonstrated an immunosuppressive microenvironment in PDAC with no prognostic value identified as an independent factor. (**A**,**B**) Compared with those in the adjacent normal samples, the densities of CD3^+^ T cells and CD8^+^ cytotoxic T cells in the tumor samples were significantly reduced (*p* < 0.01). (**C**,**D**) There were no differences in the densities of FOXP3^+^ Tregs and CD68^+^ macrophages between the adjacent normal and tumor samples. (**E**,**F**) Compared with the adjacent normal tissues, the ratio of CD68/CD3 or FOXP3/CD8 in the tumor tissues was significantly increased (*p* < 0.05). (**G**–**L**) Survival data on 84 PDAC patients were collected, including the following: different T cell (CD3^+^) densities; cytotoxic T cell (CD3^+^CD8^+^) densities; FOXP3^+^ T cell (CD3^+^FOXP3^+^) densities; exhausted, cytotoxic T cells (CD3^+^CD8^+^PD-1^+^) densities; macrophage (CD68^+^) densities; immunosuppressive macrophages (CD68^+^PD-L1^+^). There were no significant differences in the OS outcomes (*p* > 0.05) of the patients with high- or low-cell populations among the analyzed phenotypes. The median was used as a cutoff value to classify the patients into high- and low-expression groups. The error bars represent SEMs. The statistical analyses between the adjacent normal and tumor samples were conducted using Student’s *t*-tests. A log-rank test was used to determine the significance of the survival outcomes.

Initially, we comprehensively reviewed the densities of CD3^+^ T cells, CD8^+^ T cells, FOXP3^+^ Tregs, and CD68^+^ macrophages between the paired adjacent normal tissues and the tumor tissues. The CD3^+^ T and CD8^+^ T cells were significantly reduced in the tumor tissues compared to the adjacent normal tissues (Figure 2A,B), and there were no significant differences in the densities of the FOXP3^+^ Tregs or the CD68^+^ macrophages between the adjacent normal and tumor tissues (Figure 2C,D). The larger population of total CD3^+^ T cells and CD8^+^ cytotoxic T cells in adjacent normal tissues may reflect an active immune response against a large number of tumor cells, representing the initial stage of cancer cell transformation. Large numbers of cytotoxic T cells in adjacent normal tissues may represent a response to the tumor. We further evaluated the CD68^+^ macrophages to CD3^+^ T cells ratio and the FOXP3^+^ Tregs to CD8^+^ cytotoxic T cells ratio. Compared with the adjacent normal tissues, the tumor tissues showed significantly higher CD68/CD3 and FOXP3/CD8 ratios (Figure 2E,F), representing a more immunosuppressive phenotype in PDAC. Next, we determined whether the infiltration of specific T cells or macrophages in PDAC was an independent factor associated with patient survival outcomes. We calculated the densities of multiple lineage-selective biomarkers, such as CD3^+^, CD3^+^CD8^+^, CD3^+^FOXP3^+^, and CD68^+^, as well as the functional phenotypic CD8^+^PD-1^+^ and CD68^+^PD-L1^+^, in the TME of each patient. We then stratified the patients into low or high immune cell infiltration groups based on the median densities of each cell subset. We observed that high T cell or high macrophage infiltrations do not significantly associate with survival outcomes (Figure 2G–L). This result could be attributed to a lack of analysis of differential infiltration functional subpopulations and their spatial patterns, which may correlate with the predictive survival values. Overall, these results suggest that the functional subpopulations of cytotoxic T cells and myeloid subsets need to be re-defined to find the determinants of PDAC patient survival outcomes.

### 3.3. Multiplexed IHC Images Established Quantitative Assessment by Image Cytometry Analysis with Preserved TME Context Information

**Figure 3:**
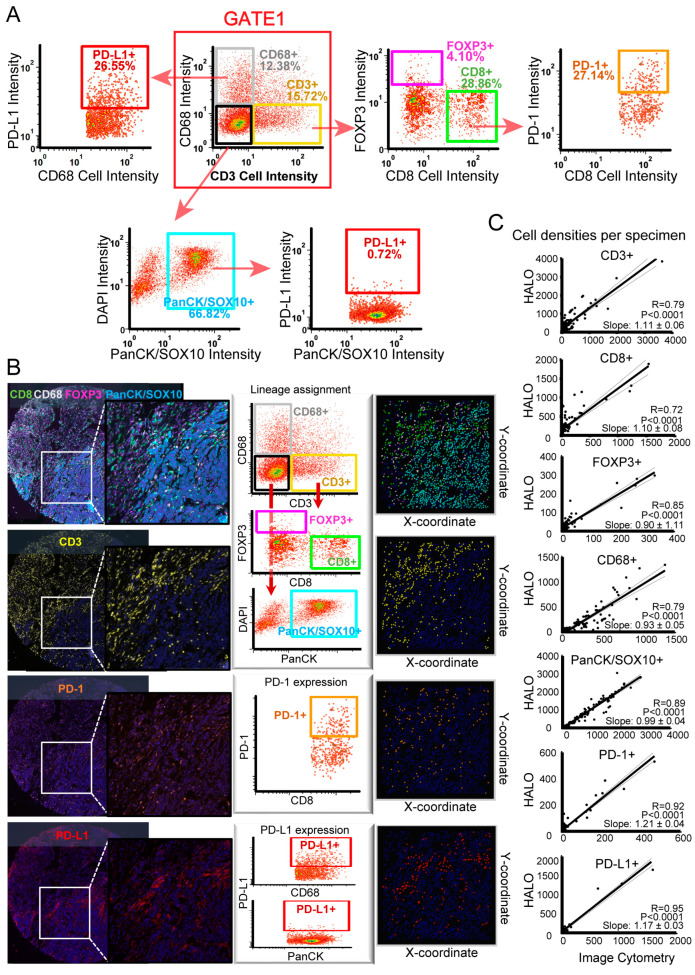
Quantitative evaluation of all parameters by image cytometry. (**A**) Image cytometry gating strategy to define the cell phenotypes. (**B**) The data were visualized after manual gating and validating the image cytometry quantification. Representative image of a TMA from a FFPE PDAC core stained by mIHC (left), with corresponding image cytometry gates (middle), and a color-coded dot plot map of the gated populations (right). (**C**) The cell densities were identified by our image cytometry gating strategies; those image cytometry methods showed robust correlation with previous ones using the HALO platform (the current standard procedure). The linear regression and the 95% confidence interval for the slope are displayed.

Similar to the flow cytometry (fluorescence-activated cell sorting [FACS]) data analyses, single-cell-based measurements, including shape, size, and pixel intensity, were performed. This approach allowed the visualization of qualitative assessments of signal intensity. The threshold for qualitative identification was determined based on the map distribution of each marker in the negative control. We developed a qualitative gating strategy for the panel to obtain quantitative data similar to the multiparameter, eight-color FACS. All cores corresponding to a single tumor sample were grouped using gates (Figure 3A). We validated these data after manual gating. The gated cells were visualized in a dot plot and correlated with the fluorescence distribution of the cells in the original image of the tissue environment (Figure 3B). For comparative analyses between the image cytometry and slice-based images, we used flow cytometry software to compare the densities of the CD3, CD8, CD68, FOXP3, PD-1, PD-L1, and tumor cells to those obtained by the phenotyping software (HALO), which is associated with the microscope platform and is the current gold standard. Both approaches showed strong positive correlations for all markers (N = 84; R = 0.72 or higher; Figure 3C). Furthermore, associations between the densities of CD8^+^ and PD-1^+^ and CD68^+^ and PD-L1^+^ were discovered (Supplementary Figure S2).

### 3.4. In Situ Leukocyte Analysis Identified the Proportion of PD-1 Expression in CD8^+^ TILs as a Risk Factor in PDAC

**Figure 4:**
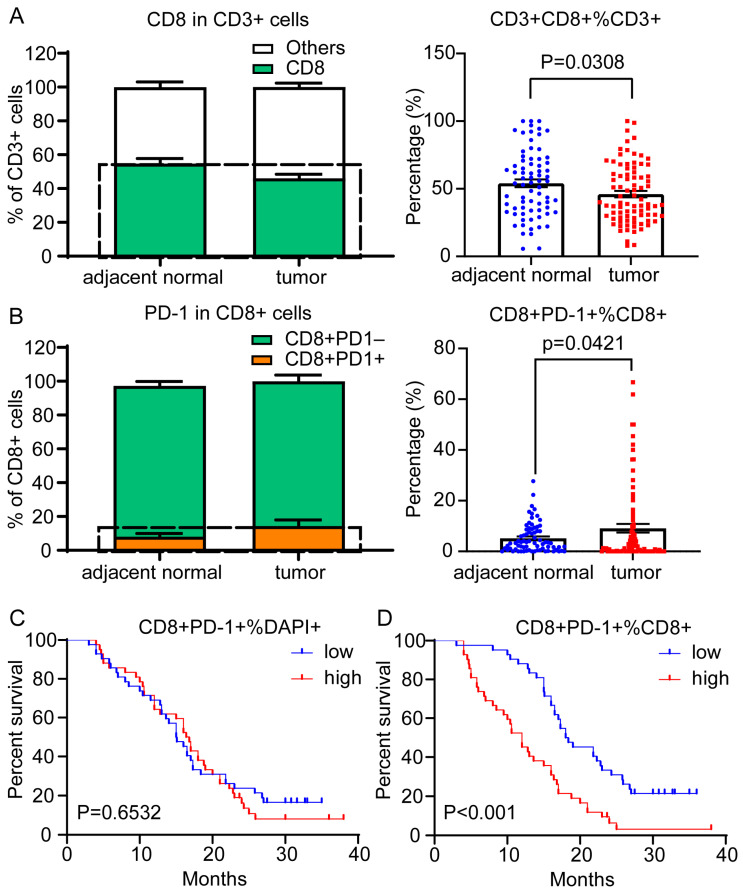
Proportion of CD3^+^CD8^+^PD-1^+^ cells among the CD3^+^CD8^+^ cells showing prognostic values in 84 PDAC patients. (**A**) Compared with the tumor tissues, the proportion of CD3^+^CD8^+^ cytotoxic T cells among the CD3^+^ T cells was significantly increased in the adjacent normal samples (*p* < 0.05). (**B**) Compared with the adjacent normal samples, the proportion of CD3^+^CD8^+^PD-1^+^ cells among the CD3^+^CD8^+^ T cells was significantly increased in the tumor tissues (*p* < 0.05). (**C**) A Kaplan–Meier curve showing no prognostic effect on the overall survival (OS) rates associated with the proportion of CD3^+^CD8^+^PD-1^+^ positive cells among all of the cells (*p* > 0.05). (**D**) A Kaplan–Meier curve illustrating a prognostic effect on the OS outcomes based on the proportion of CD3^+^CD8^+^PD-1^+^ cells among the CD3^+^CD8^+^ cells (*p* < 0.001). The median was used as a cutoff value to classify patients into high- and low-expression groups. The error bars represent SEMs. The statistical analyses between the adjacent normal and tumor samples were conducted by Student’s t-tests. A log-rank test was used to compare the survival distributions between the groups to determine significance.

Subsequently, we used multispectral staining to delineate CD8^+^ TILs in and around the tumors in the PDAC patients. The PD-1 positivity threshold was determined based on the negative control gated on the CD3^+^CD8^+^ T cells. When we looked at the entire tumor and the adjacent normal core, the proportion of CD8^+^ T cells in the CD3^+^ T cells was 54.71% on average (median = 54.5%) in the adjacent normal core and 46.08% on average (median = 39.2%; *p* < 0.001) in the tumor (Figure 4A). We further found more CD8^+^ T cells expressing PD-1 in the tumor than in the adjacent normal core (Figure 4B). Next, we further evaluated the differential infiltrating functional subpopulations of the T cells. The high proportion of PD-1^+^ CD8^+^ T cells among the total cells was not associated with patient survival outcomes (*p* = 0.6532) (Figure 4C). However, patients with a high percentage of PD-1^+^CD8^+^ T cells among the CD8^+^ T cell population had significantly poorer OS outcomes (*p* < 0.001) (Figure 4D).

### 3.5. Spatial Analysis Revealed That PD-1^+^CD8^+^ T Cells Proximal to PD-L1^+^CD68^+^ Macrophages Are Associated with Poor Prognosis

**Figure 5:**
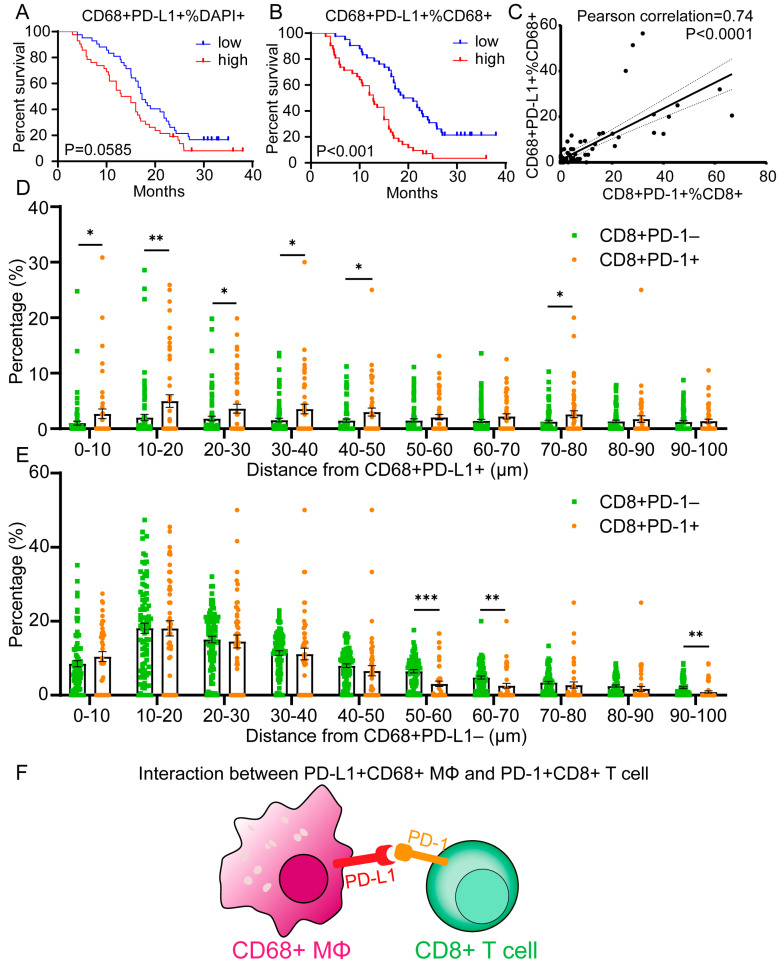
The spatial analysis revealed that the PD-1^+^CD8^+^ cells were close to the PD-L1^+^CD68^+^ cells within the TME. (**A**) A Kaplan–Meier curve showing that a low proportion of CD68^+^PD-L1^+^ among all cells tended to predict longer overall survival (OS) times (*p* = 0.0585). (**B**) A Kaplan–Meier curve illustrating the significant prognostic effect on OS based on the proportion of CD68^+^PD-L1^+^ cells among the CD68^+^ cells (*p* < 0.001). (**C**) A scatter plot showing the significant positive correlation between the proportion of PD-1^+^CD8^+^ among the CD8^+^ TILs and PD-L1^+^CD68^+^ among the CD68^+^ TAMs with the best-fit line shown. The Pearson’s correlation coefficient (r value) and the *p* value are provided at the top. (**D**) A spatial plot showing the distribution of PD-1^+^CD8^+^ cells and PD-1^-^CD8^+^ cells from the PD-L1^+^CD68^+^ cells. (**E**) A spatial plot showing the distribution of PD-1^+^CD8^+^ cells and PD-1^-^CD8^+^ cells from the PD-L1^-^CD68^+^ cells. (**F**) A diagram depicting the interaction between the PD-L1+CD68+ macrophage and the PD-1^+^CD8^+^ T cell. The median was used as a cutoff value to classify patients into high- and low-expression groups. The error bars represent SEMs. A log-rank test was used to compare the survival significance. The statistical analyses between the two groups were conducted using Student’s *t*-tests. *: *p* < 0.05; **: *p* < 0.01; ***: *p* < 0.001.

We observed a large number of PD-L1^+^ cells within the CD68^+^ macrophage population and further evaluated the functional subpopulation in the CD68^+^ macrophages. A high proportion of the PD-L1^+^CD68^+^ T cells among the total cell population demonstrated a trend of poorer patient survival outcomes (*p* = 0.0585) (Figure 5A). However, the patients with a high percentage of CD68^+^PD-L1^+^ among the CD68^+^ macrophages had significantly poorer OS outcomes (*p* < 0.001) (Figure 5B). Strikingly, we found that the proportion of PD-1^+^CD8^+^ among the CD8^+^ TILs was positively correlated with the proportion of PD-L1^+^CD68^+^ among the CD68^+^ TAMs (r = 0.74; *p* < 0.0001) (Figure 5C). In addition, the proportion of PD-L1^+^ tumor cells (PD-L1^+^ PanCK/SOX10^+^) was unrelated to the PD-1^+^CD8^+^ subset cells (Supplementary Figure S3). Therefore, we speculate that the PD-L1^+^CD68^+^ TAMs, but not the PD-L1^+^ tumor cells, are located near the specific CD8^+^ T cell subsets that exert suppressive effects. We then performed a spatial analysis and calculated the percentage of PD-1^+^CD8^+^ TILs or PD-1^-^CD8^+^ TILs per PD-L1^+^ TAMs over a range of distances from 0 to 100 μm (number of bands = 10) (Figure 5D). We found that at all of the distances studied, compared with PD-1^−^CD8^+^ TILs, the percentage of PD-1^+^CD8^+^ TILs increased significantly (0–10, 10–20, 20–30, 30–40, 40–50, and 70–80 μm; *p* < 0.05–0.01) or showed a tendency to increase around the PD-L1^+^CD68^+^ TAMs. However, more CD8^+^PD-1^-^ cells are distributed at the distances of 50–60, 60–70, and 90–100 μm from the PD-L1^+^CD68^-^ cells than the CD8^+^PD-1^+^ cells, and the PD-1^+^CD8^+^ cells showed no tendency to associate with the PD-L1^-^CD68^+^ cells (Figure 5E). Our data suggest that PD-1^+^ CD8^+^ TILs may interact closely with PD-L1^+^CD68^+^ TAMs in situ to jointly suppress potent antitumor immune responses (Figure 5F).

### 3.6. Prognostic Value Evaluation of Risk Prediction Factors

**Figure 6:**
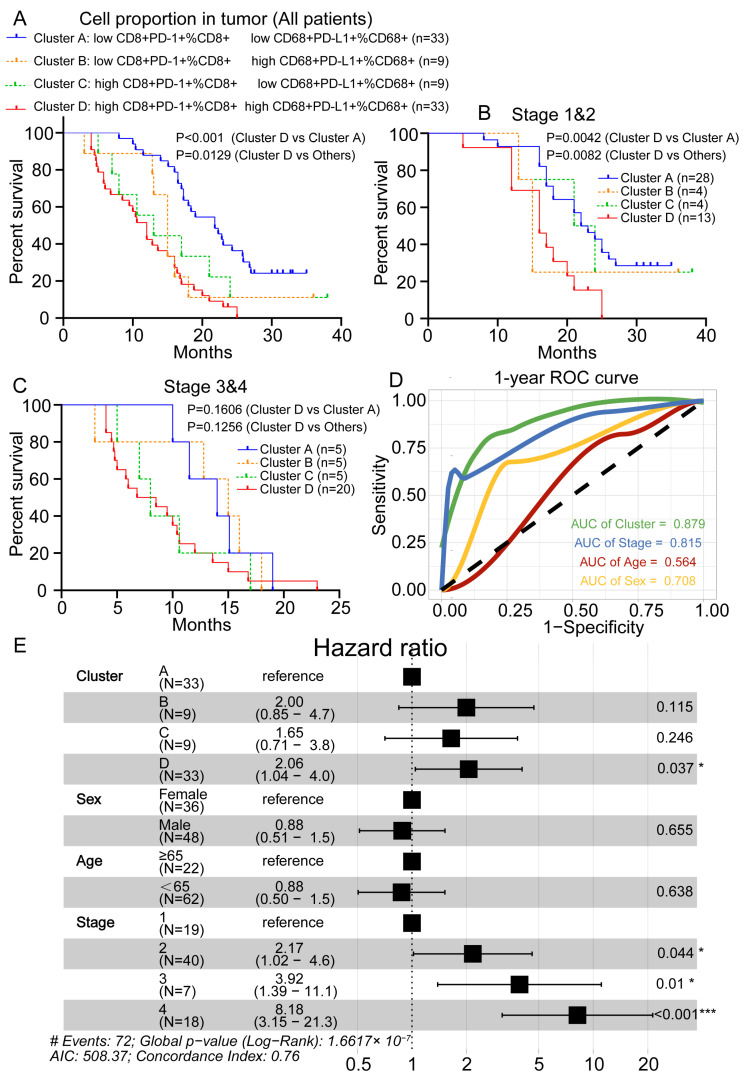
Establishment of a multivariate Cox model and assessment of its prognostic significance. (**A**) Kaplan–Meier curves were calculated to compare the overall survival (OS) outcomes and the prognostic effect in patients stratified based on the proportion of PD-1^+^CD8^+^ T cells among CD8^+^ T cells and PD-L1^+^CD68^+^ cells among CD68^+^ cells. (**B**) A Kaplan–Meier curve illustrating the significant prognostic effect in stage 1 and stage 2 patients based on patients’ stratifications. (**C**) A Kaplan–Meier curve showing no prognostic effect in stage 3 and stage 4 patients based on clusters. (**D**) Receiver operating curve analysis shows AUC values for group, age, sex, and stage in a one-year survival prediction. (**E**) An HR forest plot of the multivariate Cox model of the cluster and clinicopathological variables, including sex, age, and tumor stage. *: *p* < 0.05; ***: *p* < 0.001.

The correlations between the immune markers and the OS outcomes were assessed separately in the adjacent normal and tumor tissues. No significant correlations were found between the single immune markers and the OS outcomes, while the percentages of CD8^+^ T lymphoid cells expressing the T cell exhaustion marker PD-1 or the percentages of CD68^+^ myeloid lineages with high expression of the checkpoint PD-L1 were significantly associated with a poor outcome (*p* < 0.001). In particular, we compared the change in status (decrease or increase) of the quantitative immune markers. We considered both the PD-L1^+^CD68^+^ TAMs and the PD-1^+^CD8^+^ T cell infiltration and divided the patients into four groups based on the proportion of PD-1^+^CD8^+^ cells in the CD8^+^ T cells (high/low) and PD-L1^+^CD68^+^ cells in the CD68^+^ cells (high/low). The survival curves revealed that the patients with high CD68^+^PD-L1^+^ TAM and PD-1^+^CD8^+^ T cell proportions in the respective cell subsets had poor survival rates (Figure 6A). We further investigated different clusters associated with different cancer stages, and the results indicated that the clusters had prognostic values only in early-stage (1–2) patients and not in advanced-stage (3–4) patients (Figure 6B,C). In addition, compared with the traditional age, sex, and tumor-stage classification method, this new classification method was more accurate in predicting the one-year survival rate of PDAC patients (Figure 6D). Multivariate analyses incorporating clinical parameters revealed that a high level of infiltrating PD-L1^+^CD68^+^ within the CD68^+^ macrophage subset combined with the proportion of PD-1^+^CD8^+^ cells in the CD8^+^ T cell subset was an independent factor associated with a high risk of cancer-related death in PDAC patients (*p* = 0.037; hazard risk (HR): 2.06; 95% CI [1.04–4.0]) (Figure 6E). In conclusion, our results suggest that PD-L1^+^CD68^+^ TAMs in the TME can attract immunosuppressive PD-1^+^CD8^+^ T cells, exhibit prognostic values, and are more accurate than the results from traditional classification methods in PDAC.

## 4. Discussion

Multiplex IHC techniques have emerged as an effective approach for studying cancer, allowing the simultaneous detection of multiple markers in a single tissue section and the comprehensive study of cell phenotypes, cellular functions, and cell–cell interactions [21,22]. The evaluation of the TME using current cell markers and cell density-based assays cannot assess single-cell-based phenotypes with multiple lineage selectivity, functional status, or tissue contextual information. The strategy of multiplex IHC combined with computational imaging and multiparametric flow cytometry quantification allowed us to assess multiple lineage-selective and functional phenotypic biomarkers in the TME. Here, we defined the expression and distribution of PD-L1 and PD-1 in non-malignant cells in the PDAC microenvironment. We developed and employed analytical methods to quantify the relative proportions and locations of cells expressing PD-L1 and PD-1 and the spatial relationships between specific cell populations. We found that the percentage of lymphoid CD8^+^ T cells expressing the T cell depletion marker PD-1 or the percentage of myeloid lineage CD68^+^ macrophages with high expression of the checkpoint PD-L1 were associated with a poor prognosis. Furthermore, PD-1^+^CD8^+^ T cells proximal to PD-L1^+^CD68^+^ macrophages were associated with a poor prognosis in PDAC patients. We demonstrated the function of PD-1 cells proximal to PD-L1 cells and characterized the PD-1/PD-L1 axis, which increases the impact of immunosuppressive cellular components and affects the survival of patients with PDAC.

Despite intensive immune cell heterogeneity studies in the TME, the in situ distribution of different populations in PDAC remains unclear [23]. There is growing evidence that using marker combinations would be a more reliable method of distinguishing cell populations or activation states. Advanced techniques for studying immune cell populations exist, including IHC, CyTOF, flow cytometry, and single-cell sequencing [24,25]. However, in situ distribution information is lacking, and the multiplex IHC used in this study allows for the study of marker co-expression and spatial parameters at single-cell resolution [21]. In addition, these tissue-sectioning techniques can be analyzed similarly to flow cytometry to assess the intensity of the signal, usually using image analysis software to evaluate the output parameters of the mean intensity of fluorescence (mIF) assays. The results can be processed in a machine-readable format based on each cell and exported to the software for further analysis. In this study, we determined beforehand whether the mIHC data could be analyzed using previously defined flow cytometry workflows while maintaining the spatial information provided by this emerging technology through image cytometry. We quantified specific cell subpopulations in slides stained with mIHC using flow cytometry dot plots and associated gating strategies, as reported in previous studies [26], and found that the multispectral mIHC technique is better suited for image cytometry analysis than some other techniques for imaging cells. Based on this qualitative gating strategy, previous researchers observed that image cytometry and flow cytometry data performed similarly in terms of lymphocyte quantification [27]; thus, this imaging method can serve as a platform for multiparametric assessments of various cell lineages and realize tumor localization information. While this approach enables lineage identification based on multiple lineage selectable markers (Figure 3), its diversity is limited due to the availability of limited lineage biomarkers in specific cell types.

Having achieved this image cytometry analysis, we turned our attention to the parameters provided by mIHC and related slide-imaging systems, which are superior to the capabilities of traditional IHC methods, in which the density is assessed using multiple labels on a single slide. We also determined spatial information at the single-cell level and performed a quantitative assessment of marker co-expression in individual cells. Nonetheless, a growing number of published reports show that the density and location of specific cellular phenotypes within the TME are correlated with the proximity of PD-L1-expressing TAM or tumor-expressing PD-L1 cells [28,29]. Our study shows that the percentage of lymphoid CD8^+^ T cells expressing the T cell exhaustion marker PD-1 or the percentage of myeloid lineage CD68^+^ cells expressing the checkpoint PD-L1 are associated with a poor prognosis. The prognostic value of these methods is more important than lymphoid and myeloid cell density analyses. We believe this is because the % positive rate calculation is a ratio (the number of positive cells/total cells or a subset of cells with a positive marker function) instead of an absolute number (the number of positive cells). Therefore, this value is unlikely to change due to TME heterogeneity between different sections and/or potential sectioning artifacts or challenges in macrophage membrane segmentation. Another contributory factor may be that PD-L1^+^ macrophages can be identified through machine learning algorithms for higher reproducibility, as PD-L1 expression on the membrane may contribute to improved membrane segmentation and associated macrophages. Some of the possible strategies for improving macrophage membrane segmentation in follow-up studies include adding a stain to highlight the cell membrane to help the machine learning algorithms segment and/or segment macrophages from other immune cells in the TME.

In this study, tumor cells were also present in a local microenvironment enriched with PD-L1. This polarization may increase the local reservoir of PD-L1 available to bind PD-1 and enforce T cell suppression in the vicinity of tumor cells. However, the density of PD-L1^+^ TAMs and PD-1^+^ T cells was consistently higher. We used cell proximity analyses to determine that the spatial organization of T cells and TAMs is associated with PD-1/PD-L1 expression. To the best of our knowledge, this is the first demonstration of an unbiased classification in which closely related outcomes were observed based on quantifiably distinct cellular components and spatial organization. These results raise the possibility of characterizing additional subtype classification methods and defining spatially resolved immune signatures. Such quantifiable features may prove useful both for diagnosis and for patient stratification within diagnostic categories for therapeutic purposes.

In most of the tumors in our study, the majority of PD-L1-expressing immune cells are TAMs. This result is consistent with the observation that TAMs highly express PD-L1 in tumor tissues. Furthermore, we found that TAMs are not randomly distributed. In contrast, PD-1^+^CD8^+^ T cells were closer to PD-L1^+^ TAMs than PD-1^-^CD8^+^ T cells. Overall, our findings suggest a representation model in which the tumor’s inflammatory microenvironment is highly organized with PD-1^+^ T cells close to PD-L1^+^ TAMs and enhances immunosuppression (Figure 5). This effect may be induced by the local cytokine environment, but whether the expression on TAMs is directly dependent on the presence of tumor cells is unknown [30]. Macrophages exhibit a phenotype that is markedly plastic to their environment, and the induction of PD-L1 can include the mediation of interferon-γ and granulocyte-macrophage colony-stimulating factors (GM-CSFs), which tumors regulate alongside other pro-inflammatory cytokines [31]. Producer cells, including T cells, natural killer cells, and myeloid cells within the TME, will be analyzed in future studies. In this regard, the inflammatory TME of tumors, in which PD-L1 is expressed by non-malignant cells, including macrophages, is prominently regulated by the local microenvironment.

We examined the PD-1 expression of T cells and found high or moderate levels in CD8^+^ cells. Previous studies have determined that T cells with moderate or low levels of PD-1 expression are antigen-experienced, while those with the highest levels of PD-1 in the periphery have an irreversible “depleted” phenotype and are poised for reactivation [32]. Our data suggest that many PD-1 T cells within the tumor TME have a PD-1 phenotype ready for reactivation. While PD-1 primarily recognizes distinct T cell populations, CD68 and PD-L1 appear to identify a common TAM population. Similar to the examination of PD-L1 in the TME, we found that TAMs, but not tumor cells, promote the total microenvironmental pool of CD68 to a greater extent, which can be used to engage PD-1-positive T cells in the vicinity of tumor cells. Overall, these data provide further evidence that tumor cells reside in a specialized, microenvironmental, and immune-privileged niche in which they can exploit the coexisting escape pathways of the CD68/CD8 and PD-L1/PD-1 axes.

## 5. Conclusions

In summary, we revealed that the high PD-1^+^ expression among CD8^+^ T cells and the high PD-L1^+^ expression among TAMs are associated with each other and correlate with a poor prognosis in PDAC patients. Thus, we identified an immune “neighborhood” in which PD-L1-expressing TAMs are surrounded by high numbers of exhausted, cytotoxic T cells, playing a crucial role in immune escape. These findings also indicate that the PD-1/PD-L1 axis affects the survival of patients with PDAC.

## Data Availability

The authors declare that the main data supporting the findings of this study are available within the article and Supplementary Information. The data presented in this study are available upon request from the corresponding author.

## Supplementary Materials

The following supporting information can be downloaded at: https://www.mdpi.com/article/10.3390/cancers15051389/s1., Figure S1: H&E results of the 170 core TMA. Figure S2: Significant associations between the CD8 and PD-1 and the CD68 and PD-L1 densities. Figure S3: The proportion of PD-L1^+^ tumor cells (PD-L1^+^ PanCK/SOX10^+^) was unrelated to the proportion of PD-1^+^CD8^+^ cells in the CD8^+^ subset. Table S1: Clinical sample information. Table S2: The patients’ basic characteristics.
