# Supplementary material for: PD-1+CD8+ T Cells Proximal to PD-L1+CD68+ Macrophages Are Associated with Poor Prognosis in Pancreatic Ductal Adenocarcinoma Patients
Source: Cancers (Basel). 2023 Feb 22;15(5):1389. doi: 10.3390/cancers15051389 (PMC10000394; doi:10.3390/cancers15051389)
Supplement: Supplementary file 1 [file cancers-15-01389-s001.zip › supplementary Figure S2.pdf]

Supplementary Figure S2

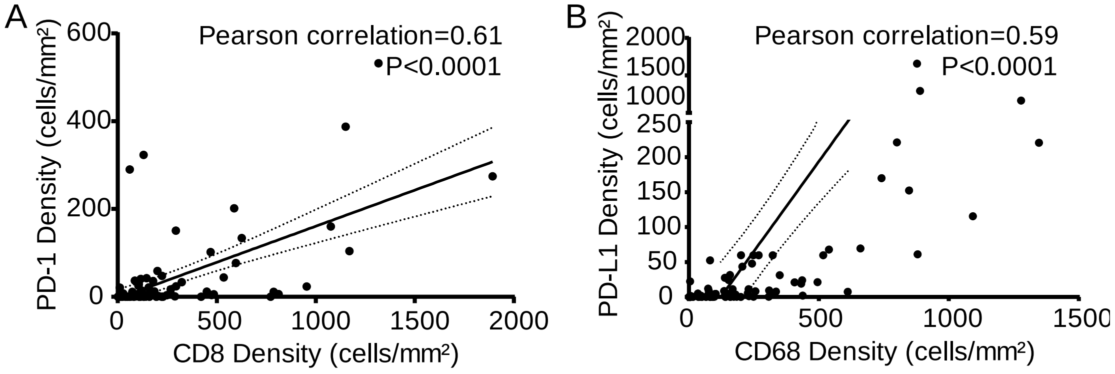

Figure S2 A,B

Figure S2. Significant associations between the CD8 and PD-1, and the CD68 and PD-L1 densities. Pearson correlation between densities of **(A)** CD8 andPD-1, **(B)** CD68 and PD-L1 (n=84 for tumor tissue samples).
