# Supplementary material for: PD-1+CD8+ T Cells Proximal to PD-L1+CD68+ Macrophages Are Associated with Poor Prognosis in Pancreatic Ductal Adenocarcinoma Patients
Source: Cancers (Basel). 2023 Feb 22;15(5):1389. doi: 10.3390/cancers15051389 (PMC10000394; doi:10.3390/cancers15051389)
Supplement: Supplementary file 1 [file cancers-15-01389-s001.zip › supplementary Figure S3.pdf]

Supplementary Figure S3

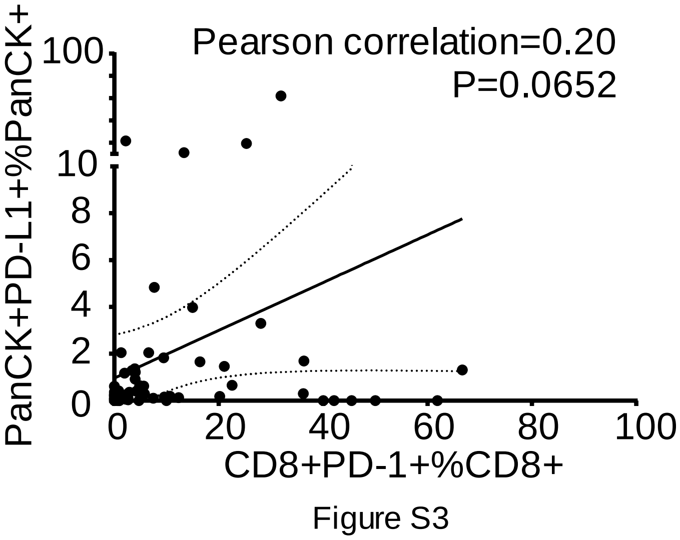

Figure S3. The proportion of PD-L1^{+} tumor cells (PD-L1^{+} PanCK/SOX10^{+}) was unrelated to the proportion of PD-1^{+}CD8^{+} cells in the CD8^{+} subset..
