# Supplementary material for: PD-1+CD8+ T Cells Proximal to PD-L1+CD68+ Macrophages Are Associated with Poor Prognosis in Pancreatic Ductal Adenocarcinoma Patients
Source: Cancers (Basel). 2023 Feb 22;15(5):1389. doi: 10.3390/cancers15051389 (PMC10000394; doi:10.3390/cancers15051389)
Supplement: Supplementary file 1 [file cancers-15-01389-s001.zip › supplementary Table S1.pdf]

Table S1. Clinical sample information.

No.	Tissues	Gender	Age	T	N	M	Stage	Survival months	Status
1	tumor	F	55	1	1	0	2	10.4	deceased
2	tumor	M	65	3	1	0	2	21.8	deceased
3	tumor	M	63	3	1	0	2	22.3	deceased
4	tumor	M	37	2	0	0	1	23.7	survival
5	tumor	M	49	2	1	0	2	22.8	deceased
6	tumor	M	48	4	1	0	3	15.1	deceased
7	tumor	F	65	2	1	0	2	31.6	survival
8	tumor	M	58	2	1	0	2	24.3	deceased
9	tumor	M	59	2	0	0	1	26.8	deceased
10	tumor	F	47	1	1	0	3	10	deceased
11	tumor	F	63	3	2	0	3	16.8	deceased
12	tumor	F	33	2	2	0	3	15	deceased
13	tumor	M	52	1	0	0	1	16.5	deceased
14	tumor	M	61	1	1	0	2	16.4	deceased
15	tumor	M	61	2	0	0	1	27.5	survival
16	tumor	F	46	4	0	0	2	5.8	deceased
17	tumor	F	64	2	2	1	4	7	deceased
18	tumor	M	45	2	1	1	4	10.4	deceased
19	tumor	F	56	3	1	1	4	11.5	deceased
20	tumor	F	56	3	1	1	4	12.8	deceased
21	tumor	M	64	3	1	0	2	16.5	deceased
22	tumor	M	65	3	2	0	3	18	deceased
23	tumor	F	62	4	0	1	4	5	deceased
24	tumor	F	53	2	1	0	4	23	deceased
25	tumor	F	61	2	0	0	1	30	survival
26	tumor	M	67	2	0	0	1	12	deceased
27	tumor	M	66	2	1	1	4	17	deceased
28	tumor	M	61	4	1	0	3	16	deceased
29	tumor	M	38	2	0	0	1	32.7	survival
30	tumor	M	48	3	1	1	4	4.5	deceased
31	tumor	M	67	3	1	1	4	4.7	deceased
32	tumor	M	67	3	2	1	3	15	deceased
33	tumor	F	57	2	0	1	4	8	deceased
34	tumor	M	42	3	1	0	2	25	deceased
35	tumor	M	54	2	1	0	2	15	deceased
36	tumor	M	66	2	0	0	1	24	deceased
37	tumor	M	66	3	1	0	2	18.8	deceased
38	tumor	M	57	2	0	0	1	16	deceased
39	tumor	F	63	3	1	1	4	12	deceased

40	tumor	M	58	3	1	1	4	10	deceased
41	tumor	M	57	3	1	0	2	21	deceased
42	tumor	M	56	2	0	0	1	30.8	survival
43	tumor	M	67	1	0	0	1	20	deceased
44	tumor	M	69	2	0	0	1	15	deceased
45	tumor	F	54	2	0	0	1	38	survival
46	tumor	F	63	2	1	0	2	13	deceased
47	tumor	M	77	2	0	0	1	17	deceased
48	tumor	M	66	3	1	0	2	17	deceased
49	tumor	F	54	3	0	0	2	8	deceased
50	tumor	M	51	2	0	0	1	16	deceased
51	tumor	F	63	3	0	0	2	13	deceased
52	tumor	F	46	4	2	0	4	4	deceased
53	tumor	F	63	1	0	0	1	18.4	deceased
54	tumor	M	67	3	1	1	4	10.6	deceased
55	tumor	F	59	2	1	0	2	25.8	deceased
56	tumor	F	62	2	0	0	1	21	deceased
57	tumor	M	62	2	1	0	2	25.9	deceased
58	tumor	F	56	3	1	1	4	10.6	deceased
59	tumor	M	69	3	1	0	2	33	survival
60	tumor	F	51	4	2	0	3	14	deceased
61	tumor	M	48	4	0	1	4	8.5	deceased
62	tumor	M	66	2	0	0	1	23	deceased
63	tumor	M	63	3	1	1	4	6.8	deceased
64	tumor	M	51	2	0	0	1	36	survival
65	tumor	M	38	3	1	1	4	4	deceased
66	tumor	F	63	2	0	0	1	12.8	deceased
67	tumor	F	52	3	1	1	4	5.8	deceased
68	tumor	M	53	2	0	0	1	35	survival
69	tumor	F	40	3	2	0	3	3	deceased
70	tumor	M	58	3	1	1	4	4	deceased
71	tumor	F	66	2	0	1	4	9.5	deceased
72	tumor	F	68	2	1	0	2	18	deceased
73	tumor	M	60	3	0	0	2	16	deceased
74	tumor	M	52	3	1	0	2	21.8	deceased
75	tumor	F	49	3	2	0	3	19	deceased
76	tumor	F	70	4	0	0	2	17.3	deceased
77	tumor	F	65	4	1	0	3	13.6	deceased
78	tumor	M	65	1	0	0	1	27	deceased
79	tumor	F	63	2	1	0	2	17.3	deceased
80	tumor	F	57	3	1	1	4	6	deceased

81	tumor	M	70	3	1	1	4	4.8	deceased
82	tumor	M	58	2	0	0	1	30	survival
83	tumor	F	60	2	1	0	2	12	deceased
84	tumor	F	57	4	1	1	4	5	deceased
85	normal	F	55	1	1	0	2	10.4	deceased
86	normal	M	65	3	1	0	2	21.8	deceased
87	normal	M	63	3	1	0	2	22.3	deceased
88	normal	M	37	2	0	0	1	23.7	survival
89	normal	M	49	2	1	0	2	22.8	deceased
90	normal	M	48	4	1	0	3	15.1	deceased
91	normal	F	65	2	1	0	2	31.6	survival
92	normal	M	59	2	0	0	1	26.8	deceased
93	normal	F	47	1	1	0	3	10	deceased
94	normal	F	63	3	2	0	3	16.8	deceased
95	normal	F	33	2	2	0	3	15	deceased
96	normal	M	52	1	0	0	1	16.5	deceased
97	normal	M	61	1	1	0	2	16.4	deceased
98	normal	M	61	2	0	0	1	27.5	survival
99	normal	F	46	4	0	0	2	5.8	deceased
100	normal	F	42	2	0	0	4	18	survival
101	normal	F	64	2	2	1	4	7	deceased
102	normal	F	56	3	1	1	4	11.5	deceased
103	normal	F	56	3	1	1	4	12.8	deceased
104	normal	M	64	3	1	0	2	16.5	deceased
105	normal	M	65	3	2	0	3	18	deceased
106	normal	F	62	4	0	1	4	5	deceased
107	normal	F	53	2	1	0	4	23	deceased
108	normal	M	67	2	0	0	1	12	deceased
109	normal	M	66	2	1	1	4	17	deceased
110	normal	M	61	4	1	0	3	16	deceased
111	normal	M	38	2	0	0	1	32.7	survival
112	normal	M	48	3	1	1	4	4.5	deceased
113	normal	M	67	3	1	1	4	4.7	deceased
114	normal	M	67	3	2	1	3	15	deceased
115	normal	F	57	2	0	1	4	8	deceased
116	normal	M	42	3	1	0	2	25	deceased
117	normal	M	54	2	1	0	2	15	deceased
118	normal	M	66	2	0	0	1	24	deceased
119	normal	M	66	3	1	0	2	18.8	deceased
120	normal	M	57	2	0	0	1	16	deceased
121	normal	F	63	3	1	1	4	12	deceased

122	normal	M	58	3	1	1	4	10	deceased
123	normal	M	57	3	1	0	2	21	deceased
124	normal	M	67	1	0	0	1	20	deceased
125	normal	M	69	2	0	0	1	15	deceased
126	normal	M	47	2	1	0	1	11	deceased
127	normal	F	54	2	0	0	1	38	survival
128	normal	F	63	2	1	0	2	13	deceased
129	normal	M	77	2	0	0	1	17	deceased
130	normal	M	66	3	1	0	2	17	deceased
131	normal	F	54	3	0	0	2	8	deceased
132	normal	M	51	2	0	0	1	16	deceased
133	normal	F	63	3	0	0	2	13	deceased
134	normal	F	46	4	2	0	4	4	deceased
135	normal	F	63	1	0	0	1	18.4	deceased
136	normal	M	67	3	1	1	4	10.6	deceased
137	normal	F	59	2	1	0	2	25.8	deceased
138	normal	F	62	2	0	0	1	21	deceased
139	normal	M	62	2	1	0	2	25.9	deceased
140	normal	F	56	3	1	1	4	10.6	deceased
141	normal	M	69	3	1	0	2	33	survival
142	normal	F	51	4	2	0	3	14	deceased
143	normal	M	48	4	0	1	4	8.5	deceased
144	normal	M	66	2	0	0	1	23	deceased
145	normal	M	63	3	1	1	4	6.8	deceased
146	normal	M	51	2	0	0	1	36	survival
147	normal	M	38	3	1	1	4	4	deceased
148	normal	F	52	3	1	1	4	5.8	deceased
149	normal	M	53	2	0	0	1	35	survival
150	normal	F	40	3	2	0	3	3	deceased
151	normal	M	58	3	1	1	4	4	deceased
152	normal	F	66	2	0	1	4	9.5	deceased
153	normal	F	68	2	1	0	2	18	deceased
154	normal	M	60	3	0	0	2	16	deceased
155	normal	M	52	3	1	0	2	21.8	deceased
156	normal	F	49	3	2	0	3	19	deceased
157	normal	M	51	3	0	0	2	23.4	survival
