# Supplementary material for: PD-1+CD8+ T Cells Proximal to PD-L1+CD68+ Macrophages Are Associated with Poor Prognosis in Pancreatic Ductal Adenocarcinoma Patients
Source: Cancers (Basel). 2023 Feb 22;15(5):1389. doi: 10.3390/cancers15051389 (PMC10000394; doi:10.3390/cancers15051389)
Supplement: Supplementary file 1 [file cancers-15-01389-s001.zip › supplementary Table S2.pdf]

Table S2. Characteristics of patients.

Parameter	Stratification	Number of patients
Gender	Male	50
	Female	37
Age	＜65	22
	≥65	65
Status	Deceased	74
	Survival	13
Tumor	T1	7
	T2	38
	T3	32
	T4	10
Lymph Node	N0	34
	N1	44
	N2	9
Metastasis	M0	64
	M1	23
Stage	1	24
	2	27
	3	11
	4	25
