# Supplementary figures and images for: PD-1+CD8+ T Cells Proximal to PD-L1+CD68+ Macrophages Are Associated with Poor Prognosis in Pancreatic Ductal Adenocarcinoma Patients
Source: Cancers (Basel). 2023 Feb 22;15(5):1389. doi: 10.3390/cancers15051389 (PMC10000394; doi:10.3390/cancers15051389)

## Supplementary Figure S1

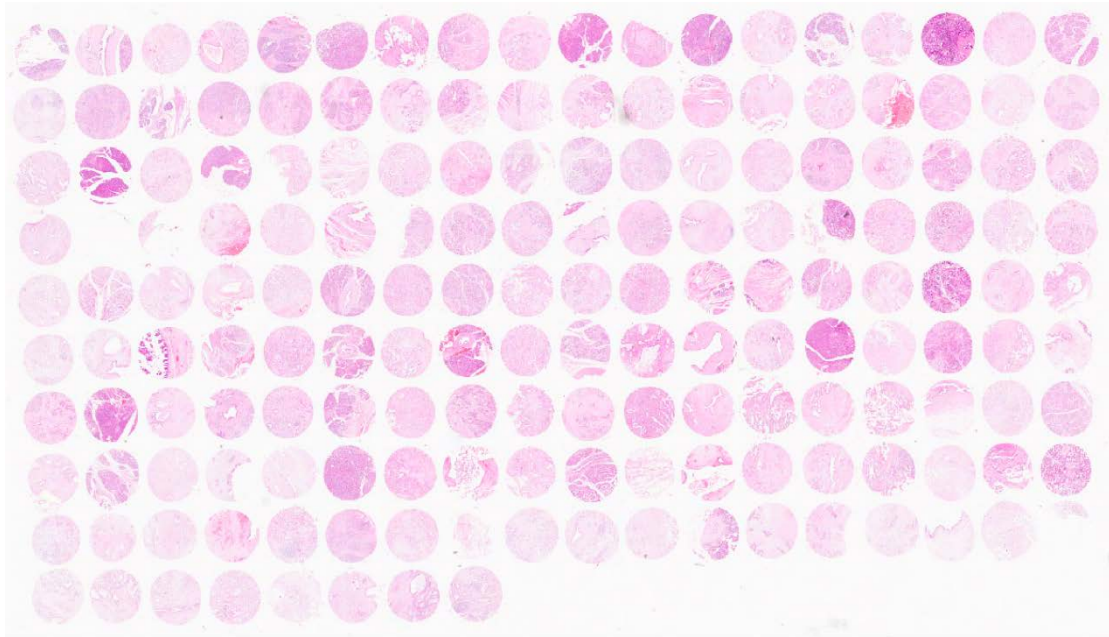

Figure S1

**Figure S1. H&E results of the 170-core TMA.**

Supplement: Supplementary file 1 [file cancers-15-01389-s001.zip › supplementary Figure S1.pdf]
